# Dose omission to shorten methacholine challenge testing: clinical consequences of the use of a 10% fall in FEV_1_ threshold

**DOI:** 10.1186/s13223-018-0309-y

**Published:** 2018-12-19

**Authors:** Valérie Lévesque, Claude Poirier, Bruno-Pierre Dubé

**Affiliations:** 1 Département de médecine, Service de pneumologie, Centre Hospitalier de l’Université de Montréal (CHUM), 1051 Sanguinet, Montreal, QC Canada; 2 Centre de Recherche du Centre Hospitalier de l’Université de Montréal (CRCHUM) – Carrefour de l’Innovation et de l’Évaluation en Santé, Montreal, QC Canada

**Keywords:** Asthma, Methacholine challenge testing, Respiratory physiology

## Abstract

**Introduction:**

In methacholine challenge testing (MCT), skipping a methacholine dose is suggested if FEV_1_ falls by < 5%. Using a larger threshold may further shorten test duration, but data supporting this hypothesis is lacking. We evaluated the safety and consequences of using a 10% FEV_1_ fall as threshold to skip the next dose of methacholine in patients undergoing MCT.

**Methods:**

We reviewed MCTs performed in our center in 2017–2018. A ≤ 10% FEV_1_ fall allowed the omission of the next methacholine dose. Patients of interest were those in which a dose was skipped after a previous FEV_1_ fall outside the usual range (5–10%, termed “skip_5–10%_”). Adverse events [AE; mild: > 1 nebulized salbutamol dose (2.5 mg) to reach basal FEV_1_, palpitations; severe: hypoxemia and/or need for medical attention or intervention] were compared in the skip_5–10%_ group and others. Regression analysis was used to identify predictors of AE.

**Results:**

208 MCTs were analysed (135 males, age 52 ± 15 years). Skip_5–10%_ occurred 111 times in 90 tests. Prevalence of AE was 5% and all were mild. Patients who developed AEs had lower FEV_1_, FVC and FEV_1_/FVC ratio, and higher lung volume values (all p < 0.05), but similar prevalence of skip_5–10%_ (36 vs. 44%, p = 0.64). Overall, MCTs in which at least one skip_5–10%_ occurred had a lower mean number of doses (3.1 ± 0.6 vs. 3.5 ± 1.3 doses, p = 0.007). Baseline residual volume was independently related to the development of AEs (OR 1.05, 95% CI 1.01–1.10, p = 0.01), but not the presence of a skip_5–10%_, even when the skipped dose directly led to the reaching of PC_20_ (OR 5.40, 95% CI 0.73–39.22, p = 0.10).

**Conclusion:**

Omitting a methacholine dose based on a ≤ 10% fall in FEV_1_ occurs frequently and has the potential to shorten test duration. AE are rare, but patients with worse baseline lung function and gas trapping are at increased risk of mild side effects.

## Introduction

Bronchoprovocation challenge is a critical tool in the diagnosis and monitoring of asthma. One of the first standardized methacholine challenge testing (MCT) protocol proposed a five-breath dosimeter protocol with a fixed initial methacholine dose followed by doubling increments in methacholine concentration until the provocation concentration causing a 20% decline (PC_20_) in forced expiratory volume in 1 s (FEV_1_) was reached or until the test was over [1]. Although safe and accurate, this protocol could be exhausting for patients and time-consuming for both patients and technicians. In the last decades, many protocol variations aiming at simplifying and shortening MCTs were proposed, such as the 2-min tidal breathing dosing method, the optional use of a higher initial dose of methacholine and the skipping of the next methacholine dose when FEV_1_ fell by < 5% of its baseline level [2]. These variations were shown to be safe, while maintaining diagnostic precision [2–5].

In order to further simplify and shorten MCTs, other methods have also been described in more recent years, such as using threefold skips in methacholine concentration [6], the use of 2-tiered protocols [7] or the early stopping of the test when PC_10_, rather than PC_20_, was reached at a methacholine dose of ≤ 1 mg/ml [8]. These changes made to the traditional MCT protocol offer the advantage of potentially decreasing test duration, while maintaining diagnostic precision and safety.

Current guidelines suggest the omission of the next methacholine dose if FEV_1_ falls by less than 5% of its previous value [2]. The use of a higher threshold for dose omission may further allow the test to be shortened and simplified. In our own center, we implemented the use of a < 10% threshold to allow the skipping of a methacholine dose in an attempt to decrease the overall duration of the test, but the safety and efficacy of this measure has not been formally studied yet. We therefore designed the following study to assess the safety and feasibility of using a 10% fall in FEV_1_ (as opposed to 5%) as a threshold to skip the next dose of methacholine in patients undergoing MCT.

## Methods

This is a retrospective observational analysis of MCTs performed in our center (Centre Hospitalier de l’Université de Montréal, Montréal, Canada) between October 2017 and May 2018. The study protocol was accepted by the local ethics board.

### Subjects

All adult subjects being referred to the pulmonary physiology laboratory for MCT testing during the study period were considered for inclusion in the study. They were referred by their attending physician (either local respiratory medicine specialist, allergist, internist or out-of-hospital general practitioner) for an elective MCT in order to evaluate respiratory symptoms and/or suspected asthma. Patients were excluded if they were unable to complete spirometry and MCT according to current guidelines [2].

### Protocol

All MCTs were performed according to the American Thoracic Society guidelines using the 2-min tidal breathing dosing protocol [2], with the exception of allowing the omission of the next methacholine dose up to a FEV_1_ fall of 10% from its previous value, rather than 5%. The initial methacholine dose which was chosen by the laboratory technician performing the test, based on each patient’s pre-test probability for asthma, FEV_1_ change after diluent inhalation and basal FEV_1_ value. Each MCT was followed by inhalation of nebulized salbutamol (2.5 mg). A second salbutamol dose was administered if FEV_1_ did not return to > 90% of FEV_1_ baseline value 10 min after the first dose. Medical files were reviewed to extract demographic data, presence of a history of asthma or atopy, use of inhaled medication and complete lung function test results. As per current guidelines, each step of the MCT lasted 5 min [2].

Patients of interest were those in which a dose was skipped after a previous FEV_1_ fall that was outside the usual range of 5% (“skip_5–10%_”). Bronchial hyperresponsiveness (BHR) was considered present when CP_20_ was ≤ 8 mg/ml.

### Outcomes

Outcomes of interest were the overall prevalence of skip_5–10%_, the prevalence of subjects in which a skip_5–10%_ directly led to the reaching of PC_20_ and adverse effects (AE). AE were categorized as mild: > 1 nebulized salbutamol dose (2.5 mg) to reach basal FEV_1_, coughing, palpitations; or severe: hypoxemia and/or any need for immediate medical attention or intervention.

### Statistical analysis

Data are presented as means (standard deviation), median (interquartile range) or n (percent), where appropriate. Comparison of variable between groups were performed using unpaired t-tests for continuous variables or Chi-squared tests for categorical variables. A multiple binary regression model was performed to identify independent predictors of the presence of AE. Sensitivity and specificity analyses of each variable of the regression model that showed an independent relationship to the presence of AE was performed, as well as receiver operating curve (ROC) analyses.

In all cases, a p-value ≤ 0.05 was used to identify statistical significance. Analyses were performed using SPSS v21 (IBM corporation).

## Results

### Population

A total of 212 MCT were reviewed, of which 208 were included for analysis. Four tests had to be excluded from the study because of the inability of the patients to adequately perform spirometry maneuvers. Baseline complete lung function tests were available for 186 patients (89%).

Table 1 describes the baseline characteristics of the study population, and the comparison between demographic and lung function testing values among patients with (n = 61) and without BHR (n = 147). Patients with BHR frequently used short-acting beta-agonist medication and had significantly lower values of FEV_1_/FVC, FEV_1_ and FVC, and higher values of RV (all p ≤ 0.05), suggesting more frequent airway obstruction and gas trapping in that group.

**Table 1:** Baseline patient characteristics

	All	BHR present	BHR absent	p
n	208	61	147	
Age	52 (15)	53 (17)	51 (14)	0.42
Male sex	135 (65)	41 (67)	94 (64)	0.65
BMI	28 (6)	29.2 (6.0)	27.5 (6.0)	0.07
History of asthma	60 (29)	22 (37)	38 (26)	0.11
Atopy	110 (53)	34 (62)	76 (52)	0.23
Pharmacological treatment				
SABA	75 (36)	30 (51)	45 (31)	0.008
LABA	2 (1)	0 (0)	2 (1)	0.37
LAMA	7 (3)	4 (7)	3 (2)	0.09
ICS	51 (25)	18 (31)	33 (23)	0.25
ICS–LABA combination	34 (16)	9 (15)	35 (17)	0.73
Lung function testing^a^				
FEV_1_/FVC	0.76 (0.07)	0.73 (0.09)	0.79 (0.06)	< 0.001
FEV_1_, l	2.82 (0.77)	2.55 (0.81)	2.95 (0.72)	0.001
FEV_1_, %	95 (14)	90 (15)	98 (13)	< 0.001
FVC, l	3.66 (0.95)	3.45 (98)	3.76 (0.92)	0.04
FVC, %	97 (13)	96 (14)	98 (12)	0.24
RV, l	1.86 (0.57)	2.01 (0.70)	1.79 (0.49)	0.02
RV, %	100 (25)	111 (33)	96 (19)	< 0.001
FRC, l	2.85 (0.68)	2.88 (0.75)	2.83 (0.65)	0.66
FRC, %	96 (19)	100 (23)	94 (16)	0.07
TLC, l	5.61 (1.03)	5.56 (1.00)	5.63 (1.04)	0.70
TLC, %	102 (13)	104 (14)	102 (12)	0.27
D_L_CO, %	94 (19)	92 (24)	96 (17)	0.24
Methacholine challenge				
Starting dose, mg/ml	1 (0.25–1)	1 (0.25–1)	1 (1–1)	0.001
PC_20_, mg/ml^b^	4.2 (1.5–6.8)	2.2 (1.1–5.3)	12.3 (10.0–14.1)	< 0.001

Data presented as mean (standard deviation), median (interquartile range) or n (percent), where appropriate

BHR is considered present when CP_20_ is ≤ 8 mg/ml

*BHR* bronchial hyperresponsiveness, *BMI* body mass index, *SABA* short-acting beta-agonist, *LABA* long-acting beta-agonist, *LAMA* long-acting muscarinic antagonist, *ICS* inhaled corticosteroid, *FEV*_*1*_ forced expiratory volume in 1 s, *FVC* forced vital capacity, *RV* residual volume, *FRC* functional residual capacity, *TLC* total lung capacity, *DLCO* diffusion capacity of the lung for carbon monoxide

^a^Data available for 186 patients

^b^Mean for the “BHR absent” group based on 16 patients. PC_20_ was > 16 mg/ml in the remaining 131 patients

### Prevalence of skipped doses after FEV_1_ decrease by 5–10% (skip_5–10%_)

**Fig. 1:**
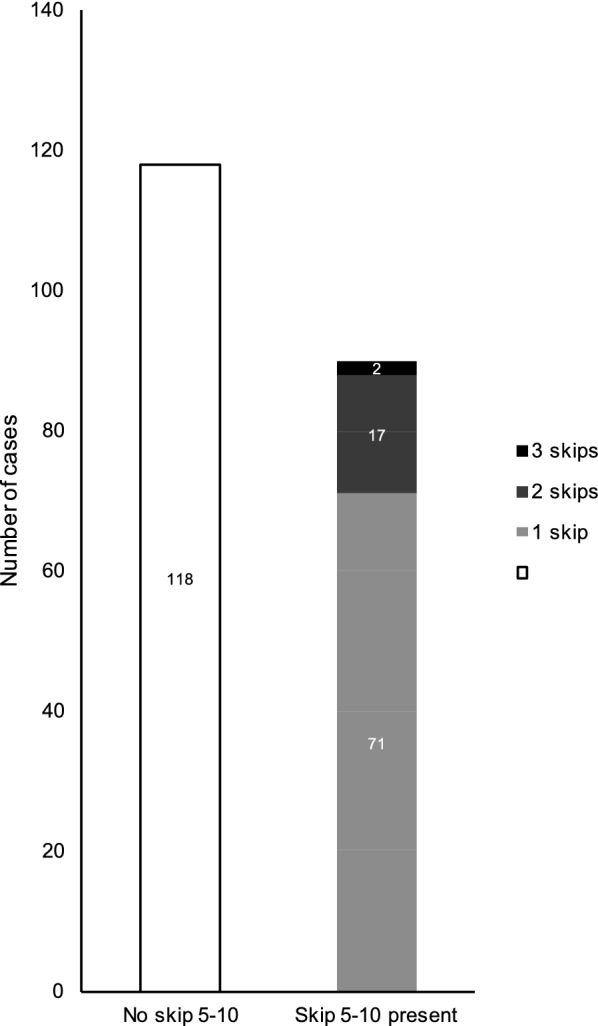
Number of patients in which a skip_5–10_ did or did not occur

Overall, there were 90 MCTs in which at least one skip_5–10%_ was performed (43%). Of these, 17 tests included 2 occurrences of skip_5–10%_ and 2 tests included 3 occurrences of skip_5–10%_, for a total of 111 skipped doses (Fig. 1). Overall, MCTs in which at least one skip_5–10%_ occurred had a lower mean number of doses (3.1 ± 0.6 vs. 3.5 ± 1.3 doses, p = 0.007). When considering the 131 subjects in which PC_20_ was not reached, this difference increased to 3.0 ± 0.1 vs. 4.0 ± 1.1 doses, p < 0.001).

### Adverse events

Across the whole study population, a total of 11 AEs were reported (5%). All AEs were mild and consisted of the need to provide patients with an additional dose of SABA after the completion of the test due to an FEV_1_ value failing to reach its baseline value, most often accompanied by cough (8/11 cases). No severe AE occurred.

Table 2 reports patient characteristics according to the development of AE. Patients in which AE occurred had significantly lower resting pulmonary function values for FEV_1_/FVC, FEV_1_ and FVC and higher values of RV and TLC. However, the mean values of FEV_1_ and FVC remained in the normal range even in this group. In addition, the presence of an AE was associated with lower PC_20_, but not with the presence of a skip_5–10%_ (36% vs. 44%, p = 0.64). However, the presence of a skip_5–10%_ that led to the reaching of the PC_20_ was more frequent in the group that presented AEs (27% vs. 8%, p = 0.03).

**Table 2:** Patient characteristics according to the presence of adverse events

	Adverse event	No adverse event	p
n	11	197	
Age	55 (14)	52 (15)	0.49
Male sex	7 (64)	128 (65)	0.93
BMI	28.3 (4.4)	28.0 (6.1)	0.87
History of asthma	5 (45)	55 (28)	0.14
Atopy	5 (45)	105 (55)	0.74
Use of any inhaled medication	8 (80)	93 (48)	0.05
Lung function testing^a^			
FEV_1_/FVC	0.71 (0.41)	0.79 (0.35)	0.02
FEV_1_, %	84 (14)	96 (14)	0.005
FVC, %	80 (29)	96 (13)	0.001
RV, %	141 (53)	98 (20)	< 0.001
FRC, %	116 (28)	95 (17)	< 0.001
TLC, %	110 (16)	102 (12)	0.06
D_L_CO, %	92 (33)	95 (19)	0.66
Methacholine challenge			
Starting dose, mg/ml	1 (0.25–1)	1 (0.25–1)	0.46
PC_20_, mg/ml^b^	2.4 (1.7)	5.5 (4.4)	0.03
Dose skipped after FEV_1_ decreased 5–10%	4 (36)	86 (44)	0.64
Dose skipped after FEV_1_ decreased 5–10% and PC_20_ reached	3 (27)	16 (8)	0.03
Number of skipped doses after FEV_1_ decreased 5–10%	0 (0–1)	0 (0–1)	0.40

Data presented as mean (standard deviation), median (interquartile range) or n (percent), where appropriate

*FEV*_*1*_ forced expiratory volume in 1 s, *FVC* forced vital capacity, *RV* residual volume, *FRC* functional residual capacity, *TLC* total lung capacity, *DLCO* diffusion capacity of the lung for carbon monoxide

^a^Data available for X patients

^b^Data available for the 77 patients with PC_20_ < 16 mg/ml

### Prediction of the occurrence of adverse events

A multiple binary regression model was performed to identify independent predictors of the presence of an AE (Table 3). Included variables were: use of any inhaled medication, baseline FEV_1_ (percent predicted), baseline RV (percent predicted) and the presence of a skip_5–10%_ that led to the reaching of the PC_20_. Of those, only RV (percent predicted) was significantly related to the development of an AE (OR 1.05, 95% CI 1.01–1.10, p = 0.01). Of note, the presence of skip_5–10%_ that led to the reaching of the PC_20_ was not an independent predictor of the presence of AE (OR 5.40, 95% CI 0.73–39.22, p = 0.10).

**Fig. 2:**
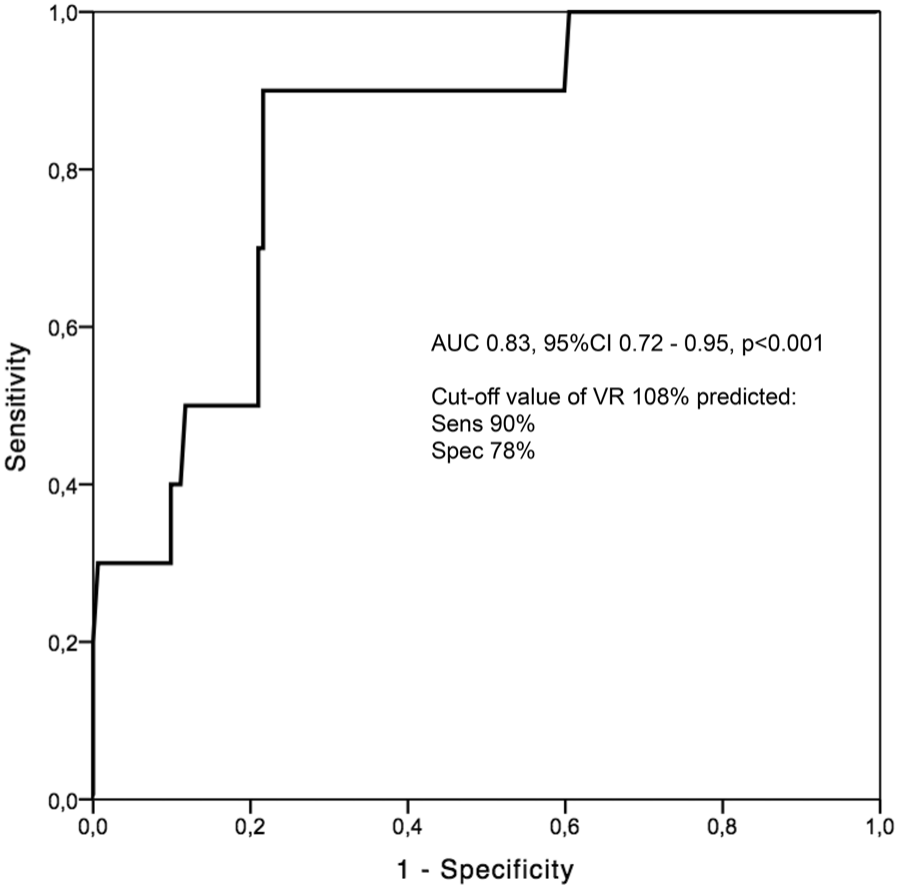
Receiver operating characteristic (ROC) curve evaluating the relationship between residual volume (at the optimal cut-off value of 108% predicted) and the presence of an adverse event during the test

A receiver operating curve analysis (Fig. 2) revealed that a resting RV (percent predicted) value of > 108% could predict the presence of an AE with a sensitivity and specificity of 90% and 78%, respectively (area under the curve 0.83, 95% CI 0.72–0.95, p < 0.001).

**Table 3:** Multiple binary regression model for the identification of predictors of adverse events

	Odds ratio	95% CI	p
Any inhaler medication	0.50	0.06–3.81	0.50
FEV_1_, *percent predicted*	0.97	0.90–1.03	0.30
RV, *percent predicted*	1.05	1.01–1.10	0.01
Dose skipped after FEV_1_ decreased 5–10% and PC_20_ reached	5.40	0.73–39.22	0.10

*FEV*_*1*_ forced expiratory volume in 1 s, *RV* residual volume

## Discussion

To our knowledge, this study is the first to evaluate the feasibility and safety of using a threshold higher than 5% in FEV_1_ fall to allow dose-omission during MCT. Our main results can be summarized as follow: (1) the use of a 10% fall in FEV_1_ threshold to skip the next methacholine dose allowed shortening the test in a large proportion of our subjects, (2) the use of this threshold was generally safe, with only mild AE being reported and (3) resting lung function values, especially RV, were associated with the occurrence of AEs.

The safety of other time-saving methods, such as increasing the initial dose of methacholine and skipping doses whenever the FEV_1_ falls by < 5% has been described several times. Troyanov et al. observed an incidence of exaggerated bronchoconstriction, defined as either a fall in FEV_1_ of > 20% after saline or > 30% after methacholine inhalation, of 10% in a group of 408 subjects, and skipped concentrations accounted for 12% of them (overall prevalence of 1%) [5]. Cockroft et al. showed that, in a population of 380 subjects undergoing MCT, 11 of them had a fall in FEV_1_ > 20% after a skipped concentration dose, but no cases of exaggerated bronchoconstriction, defined as a fall in FEV_1_ > 40%, were observed after a skipped concentration [3].

The use of rapid modern nebulizers may also be used to shorten inhalation time, time between the start of each inhalation and, eventually, total MCT time. However, these devices can be expensive, and are associated with lower PC_20_ values due to the cumulative effect of providing doses at a shorter interval [9–11].

The incidence of AEs in our study was low and comparable to the aforementioned safety data regarding the skipping of methacholine doses based on FEV_1_ fall, even though we used a larger threshold than the one of 5% usually reported.

Our finding that baseline function was related to the occurrence of AEs during the tests echoes the notion that baseline airway obstruction should help tailor certain MCT parameters (such as the methacholine starting dose) to individual patient’s characteristics [2, 12]. Our results suggest that patients with overt baseline gas trapping (as measured with RV) and those with lower FEV_1_ and FEV_1_/FVC ratio had higher rates of AEs when using our protocol, although only RV was identified as an independent predictor of the presence of AEs. In clinical practice, the usefulness of these findings may seem limited because measurements of lung volumes are not always available before performing MCT. In addition, baseline FEV_1_ in our study remained in the normal range even in the group of patients with AEs, making a pre-test risk stratification difficult. Nonetheless, these results highlight the need for clinicians to use, whenever possible, baseline lung function data to decide whether or not to proceed with a skipping protocol such as ours.

Strengths of our study include the relatively large sample size that allowed for the inclusion of a wide spectrum of patients with various pre-test probability of asthma. The majority of our subjects had no history of airway hyperresponsiveness and only half had a history of atopy, which reflects the spectrum of patients being referred to our center both by general practitioners and specialists. Selection bias was attenuated by including all tests performed during the study period, excepted for a small number of cases where patients could not complete the MCT for technical reasons.

Our study also has weaknesses that need to be acknowledged. First, the absence of a control group (one in which a standard threshold of 5% fall in FEV_1_ would have been used to skip doses) precludes a direct comparison of the prevalence of AEs between those two protocols, and the direct comparison of our results with others from the literature [5]. It remains, however, that our results support the safety and feasibility of our protocol in clinical practice. Also, the incidence of AEs was low, which may impair the statistical power of certain analyses, especially when aiming at identifying predictors of the occurrence of AEs. This low incidence of AEs may be related to the intrinsic safety of MCTs, but also to the prevalence of BHR in our cohort (29%). This prevalence is similar to some [3], but lower than other [8] studies performed in the clinical setting. Further prospective studies aiming at directly comparing the effects of using the 10% or 5% fall in FEV_1_ threshold to skip methacholine doses would help strengthen our results. In addition, studies investigating the effects of the simultaneous use of various time-saving procedures (tailoring of initial dose, threshold for dose-skipping, early termination of test) could eventually lead to an MCT protocol allowing more customization based on individual characteristics of each patient.

## Conclusion

During MCT, the prevalence of the omission of the next methacholine dose based on a ≤ 10% fall in FEV_1_ is high, and shortens test duration. When using this protocol, AEs remain rare and mild, but are associated with greater baseline airway obstruction and gas trapping. These results suggest that the use of this threshold is both feasible, useful and safe, but further studies are needed on the effect of combining various time-saving modifications to the MCT protocol.
